# Optimizing the effect of plant protease on different properties of analog cheese containing functional corn leachate

**DOI:** 10.1002/fsn3.3191

**Published:** 2023-05-17

**Authors:** Farideh Eslambeig Araghi, Rezvan Mousavi Nodushan, Afshin Jafarpour, Maryam Moslehishad

**Affiliations:** ^1^ Department of Food Science and Technology, North Tehran Branch Islamic Azad University Tehran Iran; ^2^ Department of Food Science and Technology, Garmsar Branch Islamic Azad University Semnan Iran; ^3^ Department of Food Science and Technology, Safadasht Branch Islamic Azad University Tehran Iran

**Keywords:** analog cheese, corn steep liquor, *Eryngium planum*, *Origanum majorana*, *Withania coagulans*

## Abstract

Cheese is produced in different flavors, textures, and forms by coagulating the milk protein casein. This study investigated the possibility of producing analog cheese by using corn steep liquor with *Withania coagulans* extract (WCE) and adding *Eryngium planum* extract (EPE) and *Origanum majorana* extract (OME) as functional ingredients. Different physicochemical, microbial, texture, and sensory properties of the samples were evaluated. The results obtained for moisture factor, fat, ash, water content, L*, b*, firmness, overall form, *Lactobacillus* and overall acceptance of the effect of all three process variables, pH, and acidity show that only the effect of WCE and OME is significant. Also, the protein of the samples was significant only on WCE and EPE (*p* < .001). The results showed that an increase in the levels of independent variables resulted in an increase in the amount of moisture, ash, protein, *Lactobacillus*, and b* and a decrease in fat, syneresis, texture properties, coliform, and lightness. Evaluation of the overall acceptance showed that consumer acceptance increased with the increase in WCE, but it initially increased and then decreased with the increase in EPE and OME levels. Finally, the samples containing 1.5% WCE, 1% EPE, and 0.5% OME were chosen as the optimized ones.

## Graphical abstract

The formulation of an analog cheese based on corn steep liquor was optimized. Different extracts, including WCE, EPE, and OME, were used as functional ingredients. Different physicochemical, microbial, texture, and sensory properties were evaluated.

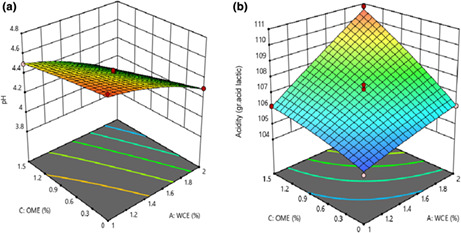

## 1 INTRODUCTION

The term “functional food” was used for the first time in Japan in the 1980s. This term became rapidly popular due to the relationship between diet and the prevention of severe and chronic diseases (Wells et al., 2017). There are different analogs or imitation dairy products containing completely nondairy alternatives (e.g., margarine and soymilk), a high quantity of dairy components, and some nondairy ingredients (e.g., vegetable fats). These days, imitation dairy products are available in many parts of the world. In general, these kinds of products are cheap and contain higher nutritional values. Besides, a wide range of functional compounds could be used in the formulation of these products (Masotti et al., 2018).

Analog cheese is a substitute or imitation of cheese in which dairy fat or protein, or both in whole or in part, is replaced by nondairy components, in particular of plant origin, such as vegetable oil, fat, and plant protein. Changing its formulation and the process conditions will affect its texture and nutritional values (e.g., reduction of salt, saturated fat, and cholesterol and enrichment with salts). Another advantage of this product is related to high textural properties during storage at refrigerator temperature (Uçar & Badem, 2016).

Sweet corn (*Zea mays* convar. *saccharata* var*. rugosa*) belongs to the Gramineae family. Corn steep liquor is a good alternative for cow's milk. Therefore, producing healthy corn steep liquor–based products, such as cheese, is an effective way to increase its consumption. In comparison to other herbal drinks, corn steep liquor has greater beneficial nutritional values as it contains high quantities of vitamins and low amounts of cholesterol and saturated fatty acids (USDA, 2010). The sweet taste and balanced aroma make this liquor superior to other vegetable‐based beverages (Aini et al., 2019).

To convert milk into clots and form clogs, various rennet and proteases (animal, microbial, and plant origins) are used during cheese production. Rennet shows a special effect as it causes milk coagulation by breaking down the Phe105‐Met 106 bond in the *k*‐casein chain, whereas other proteases are general and attack other parts of the protein chains (Forouzan et al., 2009). In addition, the plant‐based enzymes show a higher proteolytic activity; therefore, their application will improve the hard texture and poor taste of analogous cheeses (Jacob et al., 2011).

*Withania coagulans* is a plant in the Solanaceae or *nightshade* family. This medicinal plant, also called medicinal herbs, is widely used as a home remedy for curing several diseases in the Indian subcontinent and other parts of the world. This plant is a good source of dietary fibers, alkaloids, and polyphenols, so it is effective in strengthening the immune system. It shows anti‐inflammatory, antitumor, antistress, and antioxidant activities without any poisoning or side effects (Alam et al., 2012). The fruit of this plant possesses sedative, antiemetic, and diuretic properties, and is usually used as an antidiabetic agent (Hemalatha et al., 2008). This fruit also has antimicrobial, antifungal, anti‐inflammatory, antitumor, and free radical scavenging effects. It is also helpful in lowering blood lipid and sugar, preventing cardiovascular and liver diseases, and strengthening the immune system and suppressing depression (Gupta & Keshari, 2013). The plants have different medicinal properties because of the presence of various biologically active substances such as vitamins, micronutrients, minerals, and enzymes. Recently, the tendency to use herbal medicines and natural products has been increasing globally. The reason for this popularity could be attributed to the adverse effects of chemical drugs on consumer's health and environment.

*Eryngium planum* is an Asian native species of flowering plant in the Apiaceae family. This plant is used in soothing, curing hemorrhoids, and relieving rheumatic diseases, inflammation, and heartburn. Besides, its root is useful in inflammation treatment. An antiparasitic drug and intestinal worm (obtained from an eryngial incision) is produced from this plant in the United States (Khoshbakht et al., 2007). The essential oil of this plant contains monoterpenoids (71%), Sesquiterpenoids (12.6%), and diterpenoids (1.6%). The main components in *E. planum* are limonene (12.1%), beta‐sesquii (8.1%), alpha‐pinene (1.1%), and delta‐2‐carn (1.3%) (Konovalov et al, 2022).

*Origanuare majorana*, a cold‐sensitive perennial herb, contains different components such as flavonoids, tannins, steroids, and vitamins (particularly A and C). The main constituents of the essential oil (20%) are terpene 4‐al, g‐terpinene (20%), sabinin hydrate (12‐15%), e‐terpinol, sabinen, and linalool. The major phenolic compounds in this plant are synaptic acid, ferulic acid, coumarinic acid, caffeic acid, syringic acid, vanillic acid, and 4‐hydroxybenzoic acid (Charles et al., 2013).

Salehi et al. (2017) investigated the purification and characterization of a milk‐clotting aspartic protease from *W. coagulans* fruit. The proteolytic activity of this enzyme was evaluated using casein. The milk‐clotting activity was analyzed using skim milk. Mass spectrometry analysis of the purified protease and enzyme assays in the presence of protease inhibitors indicated that aspartic protease was the only enzyme responsible for milk coagulation. Furthermore, an investigation of the effect of salts on enzyme activity showed that both NaCl and CaCl_2_ reduced enzyme activity. These characteristics of the protease indicated that this enzyme could be suitable for producing low‐salt‐content cheeses.

This study aimed to investigate the possibility of producing analog cheese by using corn steep liquor with *W. coagulans* and adding the extracts of *Eryngium planum* (EPE) and *Origanum majorana* plants as functional ingredients. The optimum formulation was chosen considering the physical, chemical, microbial, and sensorial properties of the produced cheese.

## 2 MATERIALS AND METHODS

### 2.1 Materials

Milk protein concentrate (MPC) powder was purchased from MILEI Company (Germany, Baden‐wurttemberg), and whey protein concentrate (WPC) powder was obtained from Nasim Sabah Company (Iran, Mazandaran). Sweet corn (single cross variety 704) was purchased from the local market in Tehran to prepare corn steep liquor. Standard rennet of Chy‐Max (Danish dairy company Hansen) and mesophilic initiator (CHOOZIT 230, containing *lactococcus lactis* strains of Kremoris and lactis) and thermophilic initiator (532 YO‐MIX, containing *Streptococcus thermophilus* and *Lactobellus* and *Lactobacillus*) strains were used. *W. coagulans* fruits were collected from mountains located in Iranshahr (Sistan and Baluchestan Province, Iran). *E. planum* was collected from farms in Mazandaran Province (Iran). Marjoram was purchased from Shafabakhsh Medicinal Plants Company (Tehran, Iran).

### 2.2 Preparation of extracts

Corn steep liquor was prepared based on the method of Aini et al. (2017) with some modifications. First, cleaned corn kernels were mixed with lukewarm water in the ratio of 50:50 (corn:water) and homogenized for 10 min using a blender. The liquor mixture was filtered using a cleaning cloth. Then, it was purified by heating at 90°C for 5 min. The corn steep liquor was cooled and stored overnight in the refrigerator to be used in producing the analog cheese samples.

The analog cheese samples were produced based on the method of Aini et al. (2017) with some modification. MPC and WPC powders were thoroughly mixed with corn steep liquor, in a specific proportion (15% by weight/volume) with 5% oil and 0.15% of twin 80 emulsifiers, using a laboratory mixer at 60°C for 5 min. The mentioned quantities of ingredients were determined according to preliminary tests. To create a homogeneous and uniform texture, the prepared mixture was homogenized for 90 s at 2,000 rpm using a stirrer (FALK stirrer, Bergamo, Italy). Pasteurization was performed by heating the mixture at 80°C for 1 min. After the pasteurized mixture (34°C) was cooled, different percentages of *Withania coagulans* extract (WCE) (1%, 2%, and 3%) and *Eryngium planum* (EPE) and Marjoram (OME) extracts (0.5%, 1%, and 1.5%) were added. After the samples were packaged, they were coagulated by incubating at 34°C for 25 min. Salt (1.5%) was added, the packages were sealed, and the samples were stored at 37°C for 19 h and then at 5°C for 72 h.

### 2.3 Analyses of chemical properties

Acidity of cheese was measured by determining the amount of measurable lactic acid by titrating a diluted solution of cheese with a standard alkaline solution (one‐ninth normal sodium) in the presence of phenolphthalein reagent; the pH of the samples was measured using a digital pH meter (Hanna Instruments, Villafranca, Italy) by inserting the electrode of the pH meter directly into the treatments, and 5 g of the homogenized sample of cheese was taken in a container that had reached a constant weight and placed in an oven at 105–110°C to reach a constant weight. The difference in the weight of the moisture content and what remained in the container was reported as dry matter; first, the sample was burned in a special container to remove smoke, the container containing the sample was turned into ash in an electric furnace at a temperature of 550–600°C for 4–5 h, and then the amount of ash was measured; protein content was determined using an Automatic Kjeldahl Digestion Apparatus (model PDU‐500, Isfahan, Iran), and a correction factor of 6.38 was applied to calculate the a of protein; some of the cheese was crushed in a Chinese mortar. The cheese capsule was placed on the scale, and 3 g of crushed cheese was weighed, placed inside the capsule, and then transferred to the butyrometer cylinder. Ten milliliters of amylic alcohol was added to it, and it was filled with distilled water up to the graduated line of the butyrometer. The butyrometer was placed in a Gerber centrifuge (FUNK Gerber, Berlin, Germany) for 4 min, and then the fat number was read using the official methods of AOAC (2000). The fat content of the samples was measured by Gerber volumetric method using Gerber centrifuge. For carbohydrate content, 0.1 ml of the alcoholic extract was mixed with 3 ml of freshly prepared anthrone (150 mg of Antron + 100 ml of sulfuric acid, 72%). This solution was heated in a water bath for 10 min up to the reaction color change Then its absorption rate was measured using a spectrophotometer. Wavelength reading of 485 nm was obtained, and the quantity of sugar in the solution was calculated.

### 2.4 Measurement of physical properties

#### 2.4.1 Determining cheese hydration

The hydration of cheese was determined by the weight ratio of isolated whey to the initial curd (Nikoofar et al., 2013).

#### 2.4.2 Cheese color analyses

The color of the cheese samples was determined using a colorimeter. L*, a*, and b* values indicate brightness, redness, and yellowness of the samples, respectively (Cooke et al., 2013).

#### 2.4.3 Texture analyses

To analyze the texture properties (hardness, adhesiveness, cohesiveness, and elasticity) of the produced cheese, texture profile analysis (TPA) was performed using probe number 5 S/P (Jooyandeh, 2009). The probe speed was set to 1 mm/s, and the probe penetrated up to 50% of the initial height of the cheese samples (10 mm depth). The probe speed was adjusted to 2 and 1 mm/s before and after the test, respectively. This experiment was performed thrice for each sample (Table 1).

**TABLE 1:** Definition of the histometric characteristics obtained and their calculation method.

Idiom	Definition	Calculation method
Hardness (g)	The force necessary to achieve the shape change	The maximum force during the first compression period
Cohesiveness	The strength of internal bonds of food	The ratio of the level of positive force in the second peak to the first peak
Gumminess (g)	The work required to break down a semisolid food item until it is swallowed	The product of stiffness and continuity
Chewiness (g/mm)	The work required to chew solid food into a state ready for swallowing	The product of stiffness, continuity, and elasticity

### 2.5 Microbial analyses

Violet red bile agar and pour plate method were used to count coliform bacteria based on Iranian standards (Duncan, 2004). The mixed culture method was used to count the mold and yeast colonies (National Standard of Iran, 2406, Duncan, 2004). To count the *Lactobacilli*, 1 g of cheese samples was weighed and added to 3.5 ml of ringer solution. Different dilutions (2–35 times) were prepared. To count the *Lactobacilli*, de Man, Rogosa, and Sharpe agar culture medium and the last three dilutions of pour plate (2–35, 0–35, and 5–35 times) were used. Finally, the samples were incubated under anaerobic conditions at 15°C for 58 h. Then, the plates were counted (Kasimoglu et al., 2004).

### 2.6 Sensory analyses

Based on the nine‐point hedonic test, the most important organoleptic properties of dairy cheese samples (color, appearance, taste, aroma, consistency, and texture) were evaluated to study the sensory properties. All skilled panelists were employees of Pegah Lorestan Company. The temperature of all samples was the same by preserving them at room temperature (22 ± 2°C) for 30 min before starting the test. Based on the importance of each of the desired quality traits, a coefficient was considered for each one based on the IDF (International Dairy Federation) recommendations (IDF, 1). Therefore, the results related to taste and aroma (the most important factors), texture, appearance, and color were multiplied by 5, 4, 1, and 1, respectively. In total, each treatment could achieve a maximum of 100 points.

### 2.7 Statistical analysis

In this study, the Box–Behnken design was used to investigate the effect of independent variables on the quality characteristics of analog cheeses. The results obtained in this design were modeled using Design Expert software (version 7.1.6). Three‐dimensional curves were inserted to investigate the relationship between dependent and independent variables. A, B, and C indicate the percentage of added WCE, EPE, and OME, respectively.

## 3 RESULTS AND DISCUSSION

### 3.1 Properties of ingredients

The results of physicochemical properties of the used raw materials in the production of analog cheese are presented in Table 2. *Eryngium planum* extract (EPE) and *Origanum majorana* extract (OME) contained the highest quantity of antioxidants and phenolic compounds, respectively. The corn steep liquor had low protein content; therefore, protein supplements were used in the production of analog cheese.

**TABLE 2:** Physicochemical properties of raw materials used in the production of analog cheese.

Material	Antioxidant properties (μg/ml)	Phenolic compounds (mg gallic acid/g extract)	Carbohydrates (%)	Protein (%)	Fat (%)	Dry matter (%)	pH
Corn leachate	65.23	13.68	19.3	3.77	0.66	6.02	6.44
*Withania coagulans*	92.29	23.87	3.73	1.22	0.22	1.06	6.93
*Eryngium planum*	50.35	94.51	6.5	3.3	0.6	0.77	7.05
*Origanuare majoran*	1873.43	137.77	59.63	11.64	0.06	0.9	6.68

### 3.2 pH and acidity of cheese

**FIGURE 1:**
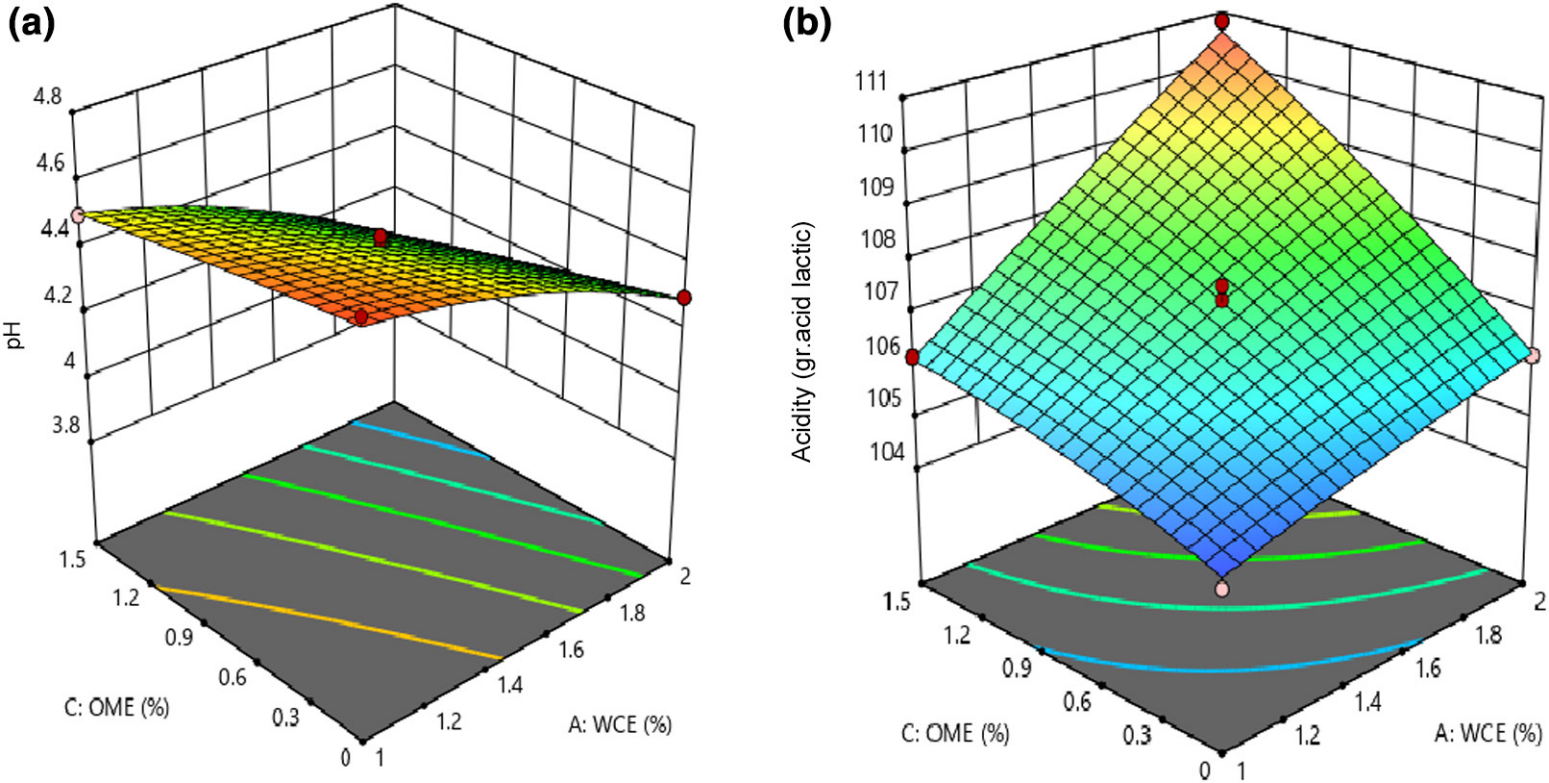
The effect of independent variables on (a) the pH and (b) acidity of analog cheese

Based on statistical analysis, the quadratic model was selected to describe the effect of independent variables on pH and acidity of cheese, and the lack of fit was not significant for these models (*p* < .05). As shown in Table 3, the effect of adding WCE, OME, and their interaction effect was significant on pH and acidity (*p* < .05). As shown in Figure 1, an increase in WCE and OME levels resulted in an increase in acidity and a decrease in pH of the sample (*p* < .05). pH and acidity are important factors influencing the stability of cheese, microorganism growth, enzyme activity, and rate of biochemical reactions during ripening. During ripening, an increase in acidity (decrease in pH) resulted in the fermentation of lactose and production of amino acids and fatty acids through proteolysis and lipolysis phenomena (Yang et al., 2017). Severe proteolysis, caused by factors such as psychrotrophic bacteria enzymes, whey, and somatic cell enzymes, can lead to the production of small peptides and high amino acids. The pH will finally increase due to the production of ammonia and amine groups after catabolism of peptides and amino acids by cheese microflora (McSweeney, 2004). An increase in acidity, as well as the plant enzyme level, could be attributed to the high number of starter bacteria in cheese. Based on the obtained results, the number of starter bacteria increased as the level of plant enzyme increased; for example, *Lactobacilli* increase the number of short‐chain peptides, free amino acids, and free fatty acids in cheese (Arenas et al., 2004). Therefore, the cheese samples with higher quantities of plant extract and starter bacteria had higher acidity. On the contrary, pH will increase by increasing the moisture content of cheese due to the decrease in H^+^ concentration (Adams & Moss, 2002). Therefore, the cheese samples with a higher quantity of plant extract showed higher pH. These were in agreement with the results obtained by investigating the coagulation effect of orange blossom on white cheese (Nasiri et al., 2020).

**TABLE 3:** Regression coefficients of responses containing pH, acidity, moisture, fat, protein, ash, and syneresis

Source	pH	Acidity (g lactic acid)	Moisture (%)	Fat (%)	Protein (%)	Ash (%)	Syneresis (%)
Model (*p*‐value)	.0001****	.0001****	.0001****	.0001****	.0037**	.0001****	.0001****
A	406.83****	362.49****	482.78****	1225.92****	37.69***	547.24****	313.25***
B	0.56^NS^	0.05^NS^	255.06****	127.36****	16.41**	102.96****	120.41**
C	93.06****	330.66****	215.56****	147.70****	0.03^NS^	26.14***	74.03*
A^2^	13.89**	13.84**	85.46****	426.71****	14.45*	3.73^NS^	1.54^NS^
B^2^	1.86^NS^	0.36^NS^	19.13**	13.65**	17.66**	24.61**	13.55**
C^2^	0.20^NS^	31.59**	0.11^NS^	2.55^NS^	1.54^NS^	35.08***	17.00**
AB	0.000^NS^	0.00^NS^	47.68***	131.29***	13.48**	6.48*	63.58**
AC	31.80**	49.39***	15.46**	122.08***	3.82^NS^	28.70**	71.01*
BC	0.50^NS^	0.01^NS^	15.84**	60.45**	2.11^NS^	37.96***	15.11*
Lack of fit (*p*‐value)	1.76^NS^	1.79^NS^	3.03^NS^	.69^NS^	3.32^NS^	4.37^NS^	3.22^NS^
*R* ^2^	0.99	0.99	0.99	0.99	0.92	0.99	0.96
Adjusted *R* ^2^	0.97	0.98	0.99	0.99	0.91	0.98	0.97
CV (%)	0.64	0.2	0.13	0.78	1.85	1.12	3.09
PRESS	0.05	3.31	0.88	0.02	4.41	0.12	2.34

*Note*: NS, *, **, *** and **** indicate insignificance, significance at *p* < .05, significance at *p* < .01, significance at *p* < .001, respectively.

Abbreviation: CV, coefficient of variance; PRESS: Predicted Residual Error Sum of Squers.

### 3.3 The changes in moisture content

**FIGURE 2:**
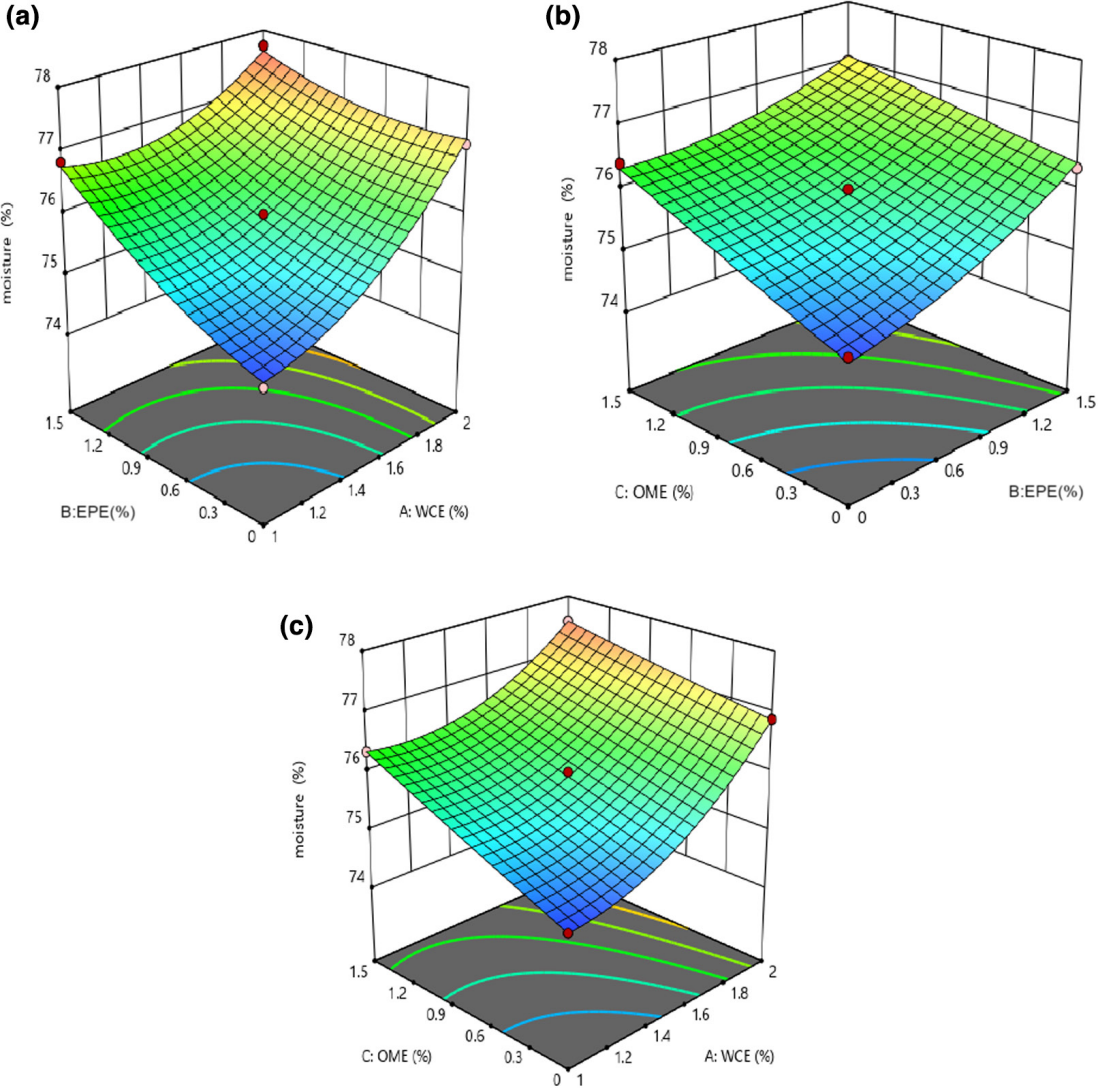
The effect of (a) EPE–WCE (*Eryngium planum* extract–*Withania coagulans* extract), (b) OME (*Origanum majorana* extract)–EPE, and (c) OME–WCE on the moisture content of analog cheese

Table 3 indicates that studying the effect of independent variables on the moisture content revealed that the fitted quadratic model was significant (*p* < .05) and the lack‐of‐fit index was insignificant for these models (*p* > .05). The effect of all variables (WCE, EPE, and OME) as well as the interaction effects of WCE–EPE and OME–EPE was significant on this parameter (*p* < .05). As shown in Figure 2, using a large number of independent variables resulted in an increase in moisture content. Pezeshki et al. (2011) reported that a higher amount of plant enzyme in the samples will cause greater proteolysis. Proteolysis increases the solubility and water absorption of proteins because of releasing polar groups such as amino and carboxyl groups of amino acids and peptides. Consequently, the higher intensity of proteolysis will result in more water absorption (Liu et al., 2020). Soodam et al. (2015) also stated that there is a direct effect between the increase in plant rennet and the moisture content in cheese. It can be therefore said that the increase in moisture content could be attributed to further proteolysis in samples containing higher quantities of plant extracts. On the contrary, the quantity of moisture content increased during cheese ripening. The quantity of milk fat inversely affected the moisture content of the samples, as Rahimi et al. (2007) and Nasiri et al. (2020) reported.

### 3.4 The fat content of cheese

**FIGURE 3:**
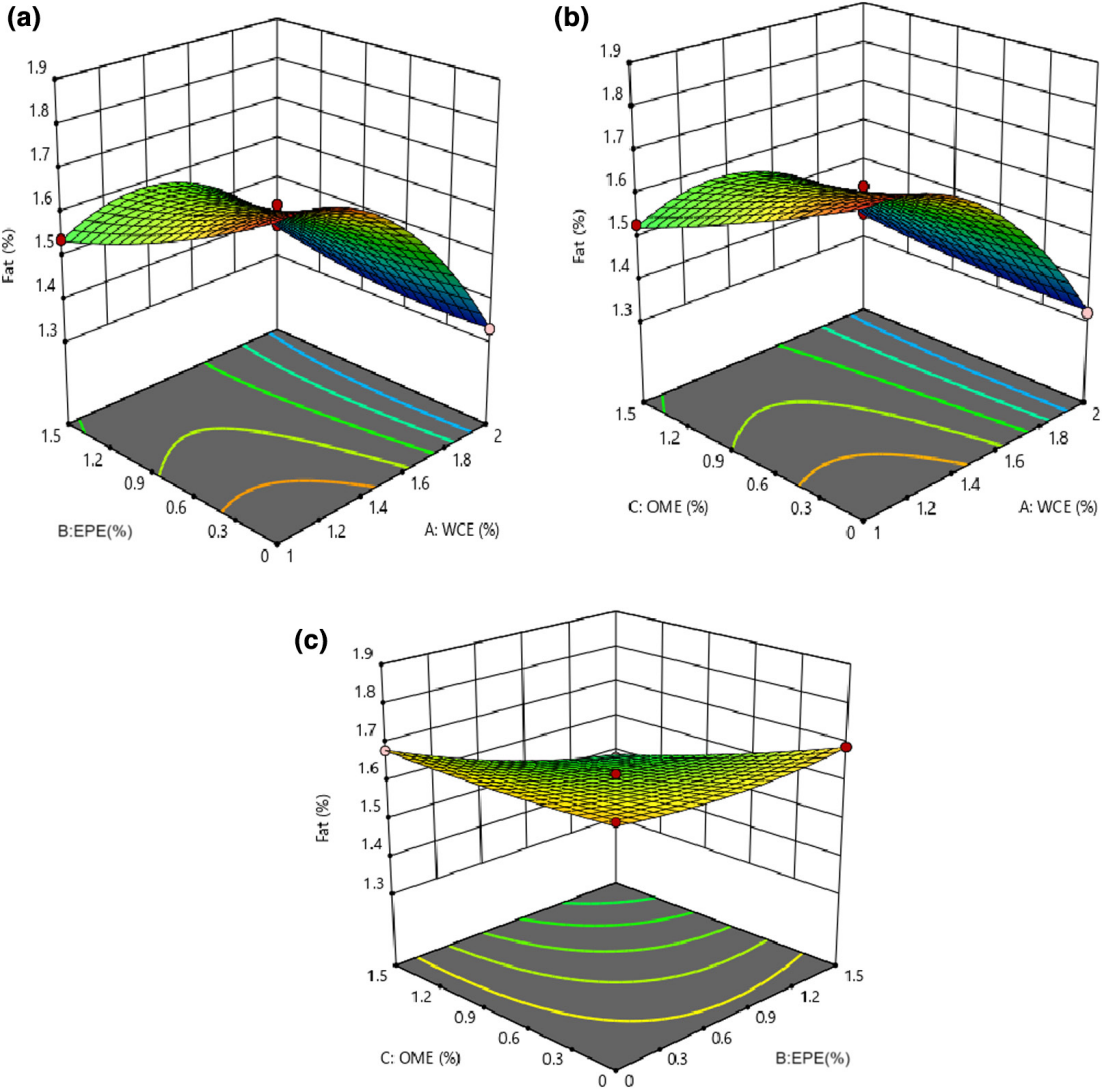
The changes in fat content of cheese versus (a) EPE–WCE (*Eryngium planum* extract–*Withania coagulans* extract), (b) OME (*Origanum majorana* extract)–WCE, and (c) OME–EPE

The results of the analysis of variance (ANOVA) showed that the fitted quadratic model was significant in studying the fat content of the samples (*p* < .05) and its lack of fit was insignificant (*p* > .05). Table 3 shows that all independent factors and their interaction effects considerably affect the change in the fat content (*p* < .05). An increase in the quantities of WCE, EPE, and OME led to a decrease in the fat content of the samples (Figure 3). Soodam et al. (2015) reported that the fat content decreased as the level of plant rennet increased. However, Pezeshki et al. (2011) stated that vegetable rennet and fungi rennet (*fromase*) significantly influence different physicochemical properties (e.g., fat content) of cheese. Processing the cheddar cheese with microbial rennet (*Bacillus subtilis*) increased its fat content in comparison to plant enzymes (Wen et al., 2021). Therefore, using proteolysis decreased the fat content of the cheese samples containing plant enzymes. In fact, fat loss is probably due to the lipolytic activity of the extract and produces many amino acids as well as increases the activity of starter bacteria. In another study, a significant difference between the fat and protein contents in colored functional yogurt indicated that the control and yogurt containing tomato extract had the highest and lowest fat content, respectively (Alirezalu et al., 2016). Rashidinejad et al. (2016) reported that adding catechin (green tea extract) had no significant effect on the fat and protein contents of cheese. O'sullivan et al. (2016) investigated the effect of adding the seaweed extract to the chemical composition of yogurt. No considerable differences were observed in fat and ash contents of control and other samples.

### 3.5 The changes in protein content

**FIGURE 4:**
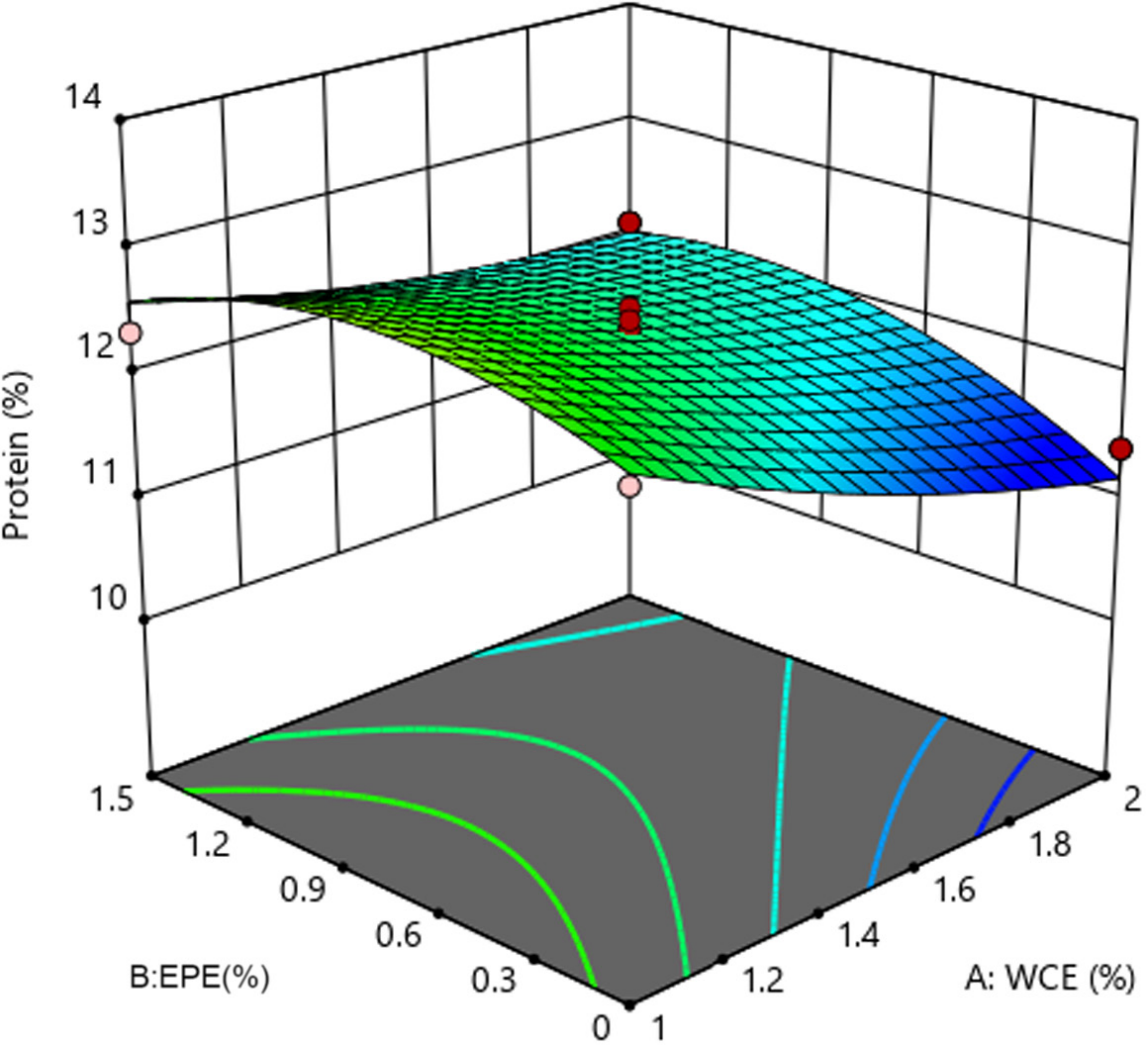
The effect of the percentages of EPE–WCE (*Eryngium planum* extract–*Withania coagulans* extract) on the protein content of the sample

Statistical analysis of protein content revealed that the fitted quadratic model was considerable (*p* < .05) and lack of fit was insignificant (*p* > .05). Table 3 shows that the linear effect of adding WCE and EPE as well as their interaction effect influenced this parameter (*p* < .05). As shown in Figure 4, the protein content of the samples decreased as the quantity of both process variables increased. However, adding EBE caused an initial increase and then a decrease in the protein content of cheese. Higher enzyme content and subsequently more proteolysis activity led to the breakdown of large molecules and peptides into amino acids and low‐molecular‐weight peptides with 12% solubility in trichloroacetic acid. These events increase the percentage of nonprotein nitrogen (Liu et al., 2020) and decrease the percentage of protein. Pezeshki et al. (2011) observed that using plant enzymes in cheese production had a greater effect on protein content reduction in comparison to fungal rennet. It can be therefore concluded that the protein content decreased due to higher proteolysis in cheese samples containing plant extracts. These results were in agreement with the reported results (Galán et al., 2008; Tejada et al., 2008).

### 3.6 Alteration in the ash content of cheese

**FIGURE 5:**
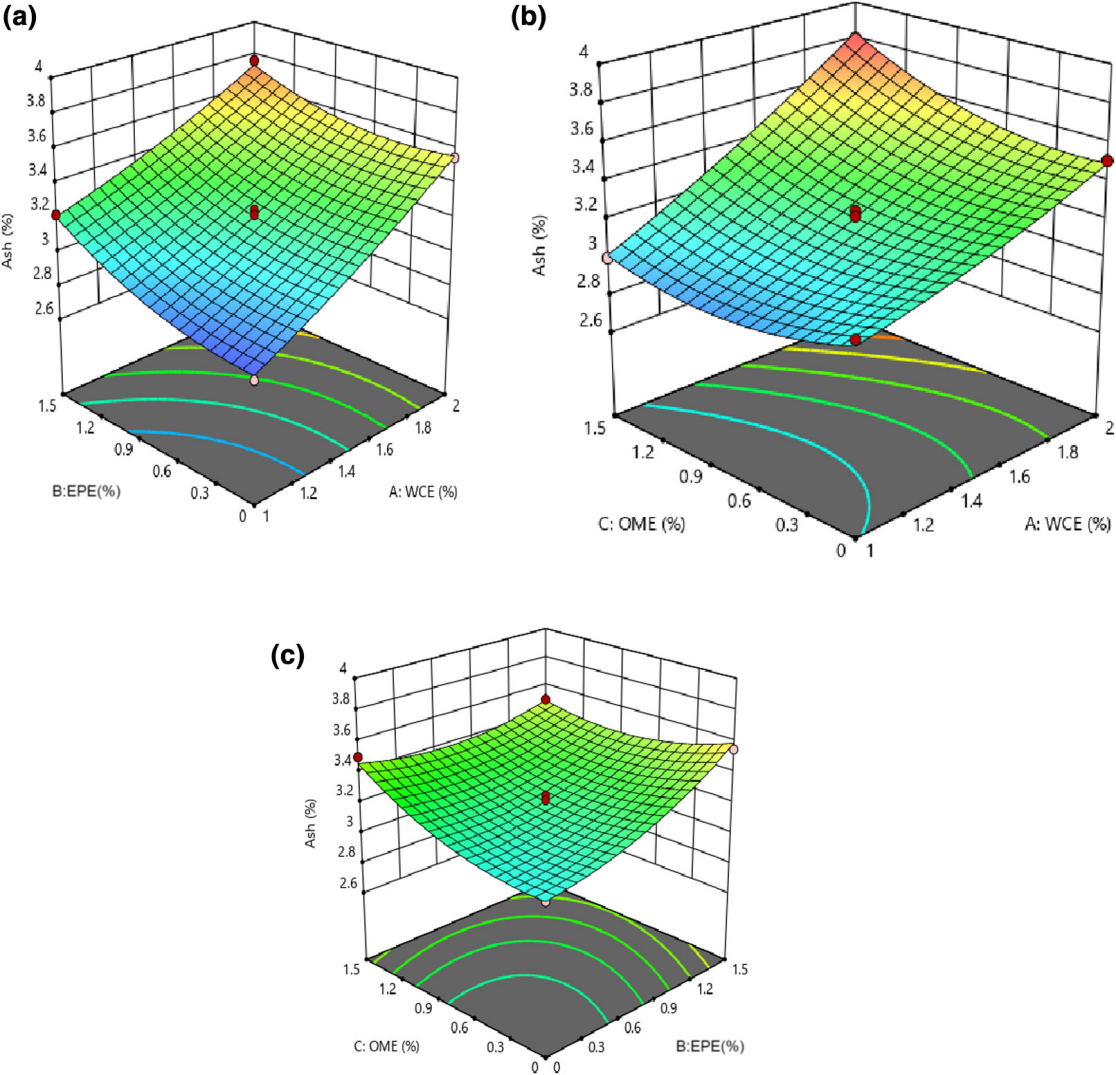
The effect of (a) EPE–WCE (*Eryngium planum* extract–*Withania coagulans* extract), (b) OME (*Origanum majorana* extract)–WCE, and (c) OME–EPE on ash content of analog cheese

The results of the ANOVA of ash content showed that the fitted quadratic model was significant (*p* < .05) and lack of fit was insignificant for these models (*p* > .05). The linear, quadratic, and interaction effects of all process variables were significant, as shown in Table 2 (*p* < .05). It was observed that increases in EPE, WCE, and OME led to an increase in the ash content (Figure 5). The ash content of cheese also reflects the presence of minerals, compounds, and free radicals in cheese (Razzaq, 2003). Using plant extracts could affect the ash content because it was unpurified and contained some minerals.

### 3.7 The quantity of syneresis

**FIGURE 6:**
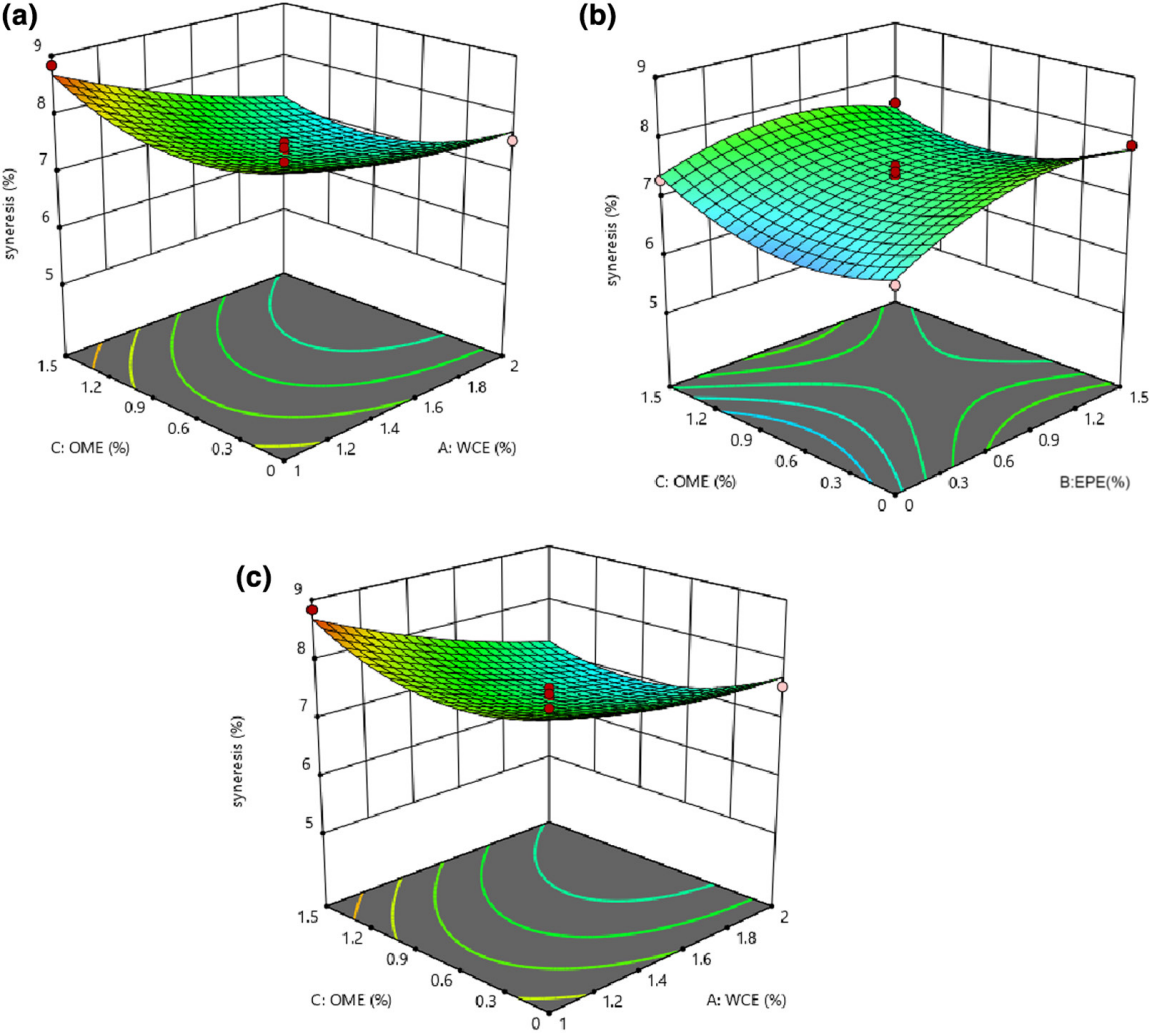
The syneresis of analog cheese versus (a) OME–WCE (*Origanum majorana* extract–*Withania coagulans* extract), (b) OME–EPE (*Eryngium planum* extract), and (c) OME–WCE

The quadratic model was consistent with the results of syneresis (*p* < .05), and the lack‐of‐fit index was not significant (*p* > .05). All linear, quadratic, and interaction effects of all independent variables were significant (*p* < .05), and only the interaction of WCE–OME was inconsiderable (*p* > .05), as shown in Table 2. The syneresis of the samples decreased as the levels of the studied variables increased (Figure 6). Syneresis of cheese is an undesirable feature during storage as it has a bad effect on consumer acceptance. Proteolysis increases the solubility and water absorption of proteins by releasing polar groups such as amino and carboxylic groups of amino acids and peptides, so higher proteolysis causes more water absorption (Liu et al., 2020). Therefore, the decrease in cheese syneresis can be attributed to the increase in proteolysis as well as water absorption. It was reported that yogurt syneresis was reduced by using extracts of blackberry and carrot (Alirezalo et al., 2015), traditional Korean plants (Joung et al., 2016), leaf of green tea and Moringa (Shokery et al., 2017), olive leaf (Esmaili, 2015), and Sheng plant (Lotfizade Dehkordi et al., 2013).

### 3.8 Changes in the color aspects of cheese

**FIGURE 7:**
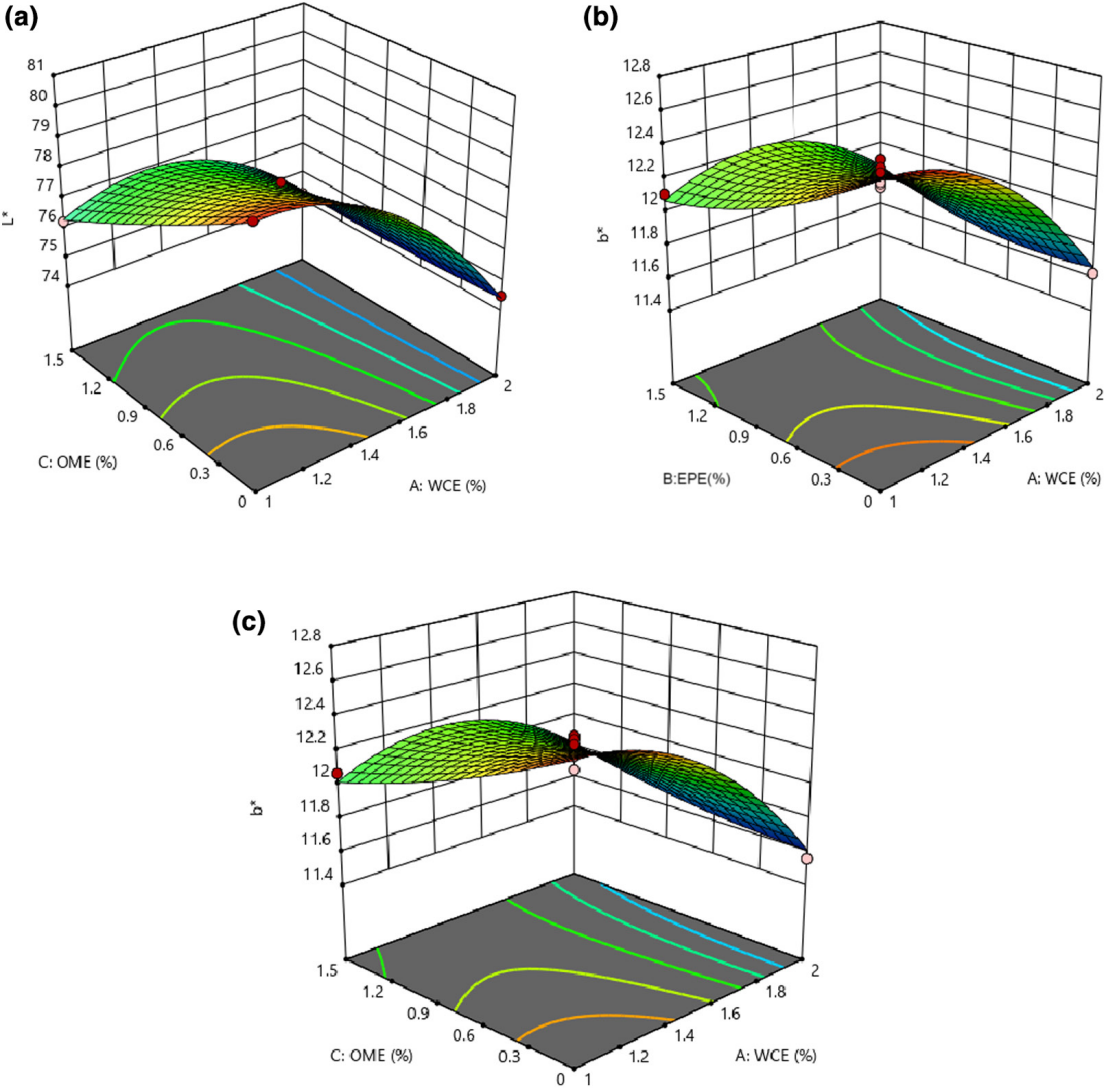
(a) L*‐value, (b) b*‐value versus EPE–WCE (*Eryngium planum* extract–*Withania coagulans* extract), and (c) b*‐value versus OME (*Origanum majorana* extract)–WCE of cheese

Based on the results of statistical analysis of L* and b* values, the fitted quadratic model and the lack of fit were significant (*p* < .05) and insignificant, respectively (*p* > .05). All linear effects and the interaction effects of EPE–OME and WCE–OME showed a significant impact on L*‐value (*p* < .05) (Table 4). The color of food products such as cheese is an important parameter affecting consumer acceptance and marketability. The color of the product is influenced by the color of the ingredients. Even though functional foods are known as health foods, lack of visual appeal to consumers results in bad marketability. Therefore, the color of the enriched products should be stable during processing and storage (Zare et al., 2011). Figure 7 shows that the amount of L* decreased with an increase in the quantity of both extracts and the plant enzyme. Changes in the protein network through proteolysis reduced this index in samples containing plant extracts. During cheese ripening, reduction in whiteness could be attributed to hydration of proteins, indicating a decrease in water content and consequently a decrease in light reflection (Rahimi et al., 2007). Wen et al. (2021) observed that the decrease in fat content caused the samples to become dull. Therefore, the reduced brightness of prepared samples with plant enzymes could be attributed to lower fat content. Unlike the b*‐value, none of the studied variables (WCE, EPE, and OME) had significant effects on a*‐value (*p* > .05). b*‐Value decreased as higher quantities of each factor were used (Figure 7; Table 4). This could be attributed to the extract that had a slightly greenish‐yellow undertone. Different biochemical reactions in cheese (e.g., Millard) will result in more yellowness to cheese color. Depending on the concentration, adding the extract of green leaf tea, Moringa leaf (Shokery et al., 2017), and olive leaf (Esmaili, 2015) reduced the brightness, increased b*‐ and a*‐values of yogurt containing green tea leaf extract, and decreased a*‐value (turning green) in yogurts containing Moringa leaf extract and olive leaf extract.

**TABLE 4:** Regression coefficients of responses containing color and texture parameters of the samples

Source	L*	a*	b*	Hardness (N)	Cohesiveness	Gumminess (J)	Chewiness (N)	Elasticity
Model (*p*‐value)	.0001****	056^NS^	.0001****	.0003***	.0038**	.0001****	.0001****	.05^NS^
A	753.77****	3.05^NS^	123.57****	54.23***	28.46**	402.65****	73.63****	0.00^NS^
B	406.26***	1.29^NS^	11.92*	60.09****	23.63**	58.43****	72.60***	1.02^NS^
C	294.11****	0.14^NS^	11.60*	33.80***	4.55^NS^	2.76***	4.32^NS^	1.00^NS^
A^2^	154.89**	0.13^NS^	46.08***	17.55**	0.26^NS^	128.2**	6.29*	0.53^NS^
B^2^	5.66^NS^	0.02^NS^	6.48*	2.06^NS^	6.66*	3.56^NS^	11.42**	0.03^NS^
C^2^	2.45^NS^	0.02^NS^	1.54^NS^	0.09^NS^	7.58*	1.33^NS^	4.96^NS^	0.05^NS^
AB	2.45^NS^	1.08^NS^	25.17***	16.90***	6.55*	39.77**	24.72**	0.00^NS^
AC	171.05***	0.00^NS^	27.23**	3.68^NS^	0.70^NS^	45.65***	4.79^NS^	0.00^NS^
BC	79.50**	2.36^NS^	10.31*	9.09*	5.33^NS^	3.96^NS^	12.62**	1.03^NS^
Lack of fit (*p*‐value)	2.56^NS^	.27^NS^	6.03^NS^	1.54^NS^	3.28^NS^	1.22^NS^	1.52^NS^	.08^NS^
*R* ^2^	0.99	0.54	0.97	0.97	0.92	0.99	0.99	0.42
Adjusted *R* ^2^	0.99	−0.05	0.94	0.92	0.82	0.98	0.98	0.34
CV (%)	0.17	22.05	0.61	5.5	6.31	3.92	2.81	18.03
PRESS	1.48	5.32	0.52	0.02	0.07	0.02	0.01	6.34

*Note*: NS, *, **, ***, and **** indicate insignificance, significance at *p* < .05, significance at *p* < .01, significance at *p* < .001, respectively.

Abbreviation: CV, coefficient of variance.

### 3.9 Cheese texture properties

**FIGURE 8:**
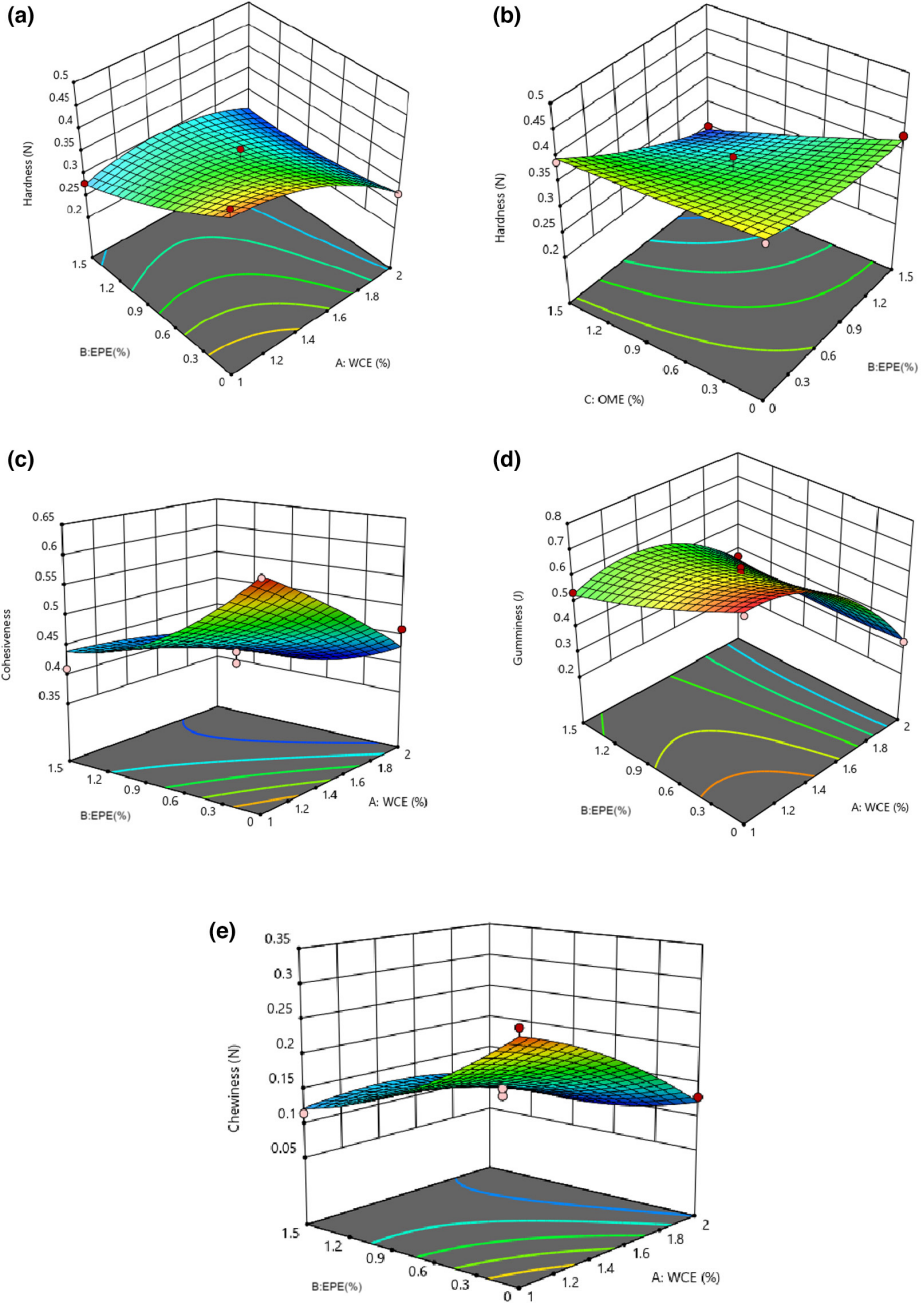
(a) Hardness versus EPE–WCE (*Eryngium planum* extract–*Withania coagulans* extract), (b) hardness versus OME (*Origanum majorana* extract)–EPE, (c) cohesiveness, (d) gumminess, and (e) chewiness of the cheese samples

The results of the ANOVA showed that the fitted quadratic model was significant for texture properties such as hardness, cohesiveness, gamminess, and chewiness (*p* < .05) and the lack of fit was not significant for them (*p* > .05). None of the independent variables had a considerable effect on the elasticity of the cheese (Table 4). As shown in Figure 8, hardness of the samples diminished linearly on increasing the WCE, EPE, and OME levels (*p* < .05). In addition, the cohesiveness, gaminess, and chewiness of the samples significantly decreased after the addition of EPE and WCE coagulant enzymes. The obtained results indicate that extracts decreased dry matter and increased the moisture content of the samples. Water molecules along with fat cells are located in the three‐dimensional network of proteins as this increase weakens the network structure and becomes more prone to rupture due to compression (Lashkari et al., 2014). Galán et al. (2008) reported that cheese produced using vegetable rennet had a softer and more creamy texture than those produced using animal rennet. This is attributed to the high proteolytic activity and breakdown of caseins, leading to the formation of soft‐texture vegetable cheese. Therefore, it can be stated that reduced hardness of these samples is caused by the higher proteolysis of refined cheese containing plant extracts. Jooyandeh (2009) reported that coagulation decreased with an increase in proteolysis during ripening because coagulation decreases and increasing the quantity of plant extract increases proteolysis. The rate of proteolysis increases with the addition of vegetable rennet (Pezeshki et al., 2011). Gunasekaran and Ak (2002) observed a lower elasticity because of higher proteolysis. Studies showed that the main reason for the reduction in tissue traits is enzymatic hydrolysis, especially the breakdown of proteins of cheese compounds. Therefore, it can be stated that higher proteolysis in samples containing plant extracts resulted in less gumminess. Ghanbari et al. (2012) produced low‐fat Iranian white cheese containing xanthan gum. They found that adding xanthan gum reduced the number of bound calcium micelles and increased repulsive power between caseins. Using the gum led to weakened bonds in the cheese structure and an increase its softness. Cooke et al. (2013) studied the effects of tragacanth gum on the rheological and functional properties of high‐fat and semi‐fat content cheddar cheese during storage. The results showed that tragacanth gum reduces the quantity of gum, hardness, and softness of cheese during storage.

### 3.10 The effect of formulation on microbial tests

The results of the ANOVA showed that the fitted quadratic model was significant for coliform and *Lactobacillus*‐containing responses (*p* < .05) and the lack of fit was not significant for these models (*p* > .05). All the variables (linear, quadratic, and interaction effects) showed significant effects on coliform growth in the produced analog cheese (Table 5). The number of coliforms decreased as higher percentages of WCE, EPE, and OME extracts were used. Jahan and Abdel‐Hakim (2017) stated that black and green cumin extracts in Domiati cheese have strong antimicrobial properties. Tejada and Fernandez‐Salguero (2003) compared the number of coliforms in two kinds of cheese produced using plant and animal rennet. They observed that the number of coliforms in processed samples using plant rennet during storage was lower; however, there was no important difference between these two cheese samples. Essential oils, extracts, and their constituents with hydrophobic properties penetrate the lipid membranes and mitochondria of bacterial cells. This disrupts the cell structures, increases their permeability, and finally results in leakage of the ions and other cell contents. The release of limited quantities of these substances is tolerable for bacteria. But cell death will eventually result due to an increase in the concentration of essential oils, thus decreasing viability and releasing large quantities of cellular contents, ions, and vital molecules (Burt, 2004). In general, the higher concentration of essential oils or phenolic substances in them causes greater antibacterial properties against food pathogens and other microorganisms. The action mechanism of these compounds includes coagulation of cell contents, disruption of the cytoplasmic membrane, proton motion, and electrical current (Lambert et al., 2001).

**TABLE 5:** Regression coefficients of responses containing microbial tests and sensory evaluation of the sample

Source	Coliform (cfu/g)	*Lactobacillus* (cfu/g)	Color	Texture	Aroma	Flavor	General acceptance
Model (*p*‐value)	.0001****	.0008**	.0081***	.0043****	.78^NS^	.72^NS^	.0001****
A	518.03****	33.83**	6.37^NS^	1.56^NS^	1.05^NS^	1.04^NS^	111.98****
B	362.92***	42.73**	112.83***	89.39***	0.67^NS^	1.74^NS^	105.65***
C	401.37****	3.54^NS^	68.60**	28.93**	0.44^NS^	0.83^NS^	94.93*
A^2^	241.38**	1.20^NS^	5.08^NS^	2.33^NS^	0.09^NS^	0.77^NS^	1.05^NS^
B^2^	162.38**	3.05^NS^	7.24*	1.98^NS^	1.77^NS^	1.94^NS^	5.64^NS^
C^2^	122.38**	2.58^NS^	1.04^NS^	1.43^NS^	0.94^NS^	0.00^NS^	2.48^NS^
AB	67.80**	15.39**	9.17*	14.62**	1.86^NS^	0.00^NS^	83.11**
AC	97.05**	0.90^NS^	1.64^NS^	0.85^NS^	1.34^NS^	0.00^NS^	4.19^NS^
BC	79.50**	5.83^NS^	22.31**	16.78**	0.56^NS^	1.94^NS^	9.66*
Lack of fit (*p*‐value)	3.37^NS^	2.78^NS^	2.19^NS^	3.78^NS^	.56^NS^	.73^NS^	4.86^NS^
*R* ^2^	0.99	0.94	0.98	0.95	0.65	0.55	0.98
Adjusted *R* ^2^	0.99	0.98	0.96	0.95	0.52	0.39	0.97
CV (%)	0.22	2.1	3.92	1.78	27.54	23.75	1.98
PRESS	0.07	0.51	0.06	0.65	7.23	5.98	0.21

*Note*: NS, *, **, ***, and **** indicate insignificance, significance at *p* < .05, significance at *p* < .01, significance at *p* < .001, respectively.

Abbreviation: CV, coefficient of variance.

As shown in Table 5, none of the process variables had significant effects on the growth of mold and yeast (*p* < .05). The results of the ANOVA (Table 5) showed that only the linear effect of WCE and EPE had a significant impact on the number of *Lactobacillus* (*p* < .05). In addition, the interaction effect between WCE and EPE was significant (*p* < .05); the number of *Lactobacillus* increased in analog cheese samples with an increase in the quantity of these variables. *Lactobacilli* are part of the microbial flora of cheese, playing an important role in its ripening (Beresford et al., 2001). This microbial group increases the number of short‐chain peptides, free amino acids, and free fatty acids in cheese. *Lactobacilli* have the ability to intensify proteolysis and improve the taste of different types of cheese (Jooyandeh, 2009).

### 3.11 Sensory evaluation

The fitted quadratic model was significant (*p* < .05), and the lack of fit was insignificant in studying sensory evaluation (*p* > .05). As shown in Table 5, the samples had a significant effect on the color and texture. As higher levels of EPE and OME were used, the acceptance of the cheese decreased. Adding higher percentages of WCE reduced color acceptance, and it caused an initial increase and then a decrease in texture scores. The results of the panel test indicated that WCE, EPE, and OME had an insignificant effect on the taste and aroma of the samples (*p* < .05). Considering the overall acceptance of the sample, consumer acceptance increased when using a higher quantity of WCE. The overall acceptance increased first and then decreased with an increase in the EBE and OME quantity.

### 3.12 Optimization of the formulation of analog cheese

In this study, the aim of optimization was to produce functional analog cheese, using *Withania* as a coagulant and EPE and OME, with the best physicochemical properties. Evaluating the formulation and process conditions showed that the optimal amounts of WCE, EPE, and OME were 1.5%, 1%, and 0.5%, respectively. Different properties of the optimized formulated cheese were evaluated, including pH (4.44), acidity (107.66 g lactic acid), moisture (74.27%), fat (1.43%), protein (11.11%), and ash (3.59%) contents as well as syneresis (6.5%), L*‐value (69.76), a*‐value (2.19), b*‐value (12.46), hardness (0.37 N), cohesiveness (0.55), gumminess (0.34 J), and chewiness (0.20 N).

## 4 CONCLUSION

The aim of this study was to investigate the analog cheese produced by using corn steep liquor with *W. coagulans* and adding EPE and OME as functional ingredients. A complete evaluation of all physicochemical, textural, microbial, and sensory properties showed that adding plant coagulation enzymes WCE, EPE, and OME significantly affected the quality of analog cheese. It was found that there was a significant effect on moisture, fat, ash, syneresis, L*‐value, b*‐value, hardness, coliform, *Lactobacillus* and overall acceptance, pH, and acidity, and only the effect of WCE and OME was significant. An increase in the levels of independent variables increased *Lactobacillus*, b*‐value, and the contents of moisture, ash, and protein and decreased fat content, syneresis, texture properties, coliform, and lightness. The sensorial evaluation showed that the overall acceptance of the samples increased as a higher quantity of WCE was used. The overall acceptance increased first and then decreased with an increase in the quantity of EPE and OME.

## ACKNOWLEDGEMENTS

All authors are most grateful and would like to express their thanks to the Editorial Board members and reviewers, for so generously giving their advice and time to reviewing the manuscript.

## FUNDING INFORMATION

The research received no external fuding.

## Data Availability

The data that support the findings of this research are available from the corresponding author upon reasonable request.
